# How Do Nurse Researchers Determine Risk When Applying for Ethical Approval for Qualitative Research?

**DOI:** 10.3390/nursrep16030091

**Published:** 2026-03-05

**Authors:** Richard Gray

**Affiliations:** School of Nursing and Midwifery, La Trobe University, Melbourne, VIC 3086, Australia; r.gray@latrobe.edu.au

Health research often requires human participants to complete one or more study-related tasks (fill out questionnaires, attend an interview, undergo an experimental treatment, etc.) requiring them to take some level of risk. Risks to research participants should be considered on a continuum from low (minor burden or inconvenience) to high (significant harm) [1]. Most research involves low levels of risk. For example, an interview-based study about nurses’ experiences of participating in a simulated learning environment would likely not be associated with risks beyond inconveniences (e.g., the time taken to meet with a researcher). Other types of research entail substantially higher levels of risk. Taking part in a clinical trial testing a new cancer treatment may involve participants potentially experiencing difficult and unpleasant side effects or even death. It is a foundational principal of ethical research that researchers understand these risks and explain them to the ethics committee or other review body to inform their decision as to whether the research should be approved [1]. It is also important for risks to be fully explained to potential participants so that they can make informed decisions about participating in the research [2].

In addition to serving as Editor-in-Chief of *Nursing Reports*, I am the Chair of a University Human Research Ethics Committee (HREC). In Australia, full review by an HREC is only required if there is a foreseeable risk that participating in a study may cause harm [1]. As a committee, we see many applications from researchers proposing qualitative studies (interviews, focus groups, and ethnography) for which full review by an HREC is determined necessary because of the risks associated with the work, with the typical case being that the research may “distress” the participants. Almost without exception, investigators do not provide evidence as to the potential risks associated with the proposed research; instead, it seems that judgements are based on professional experience and informed conjecture. Consequently, HRECs may be expending considerable effort (and resources) reviewing studies where the actual risk to participants is extremely low and, arguably, the project could be reviewed through a more appropriate expediated low-risk pathway.

One way in which researchers could make a more informed judgement about the risk rating of their research would be to review similar published studies to identify harm (or adverse events) that occurred while conducting the work. For some types of research, the importance of reporting adverse events is well established, such as in clinical trials; that said, there are significant issues with how well researchers adhere to these requirements [3]. For other research methodologies, the importance of harm reporting is seemingly less of an established convention.

I am using adverse events as proxy indicator of risk, which, at least in my mind, seems to be a reasonable assumption. That said, I would like to acknowledge that adverse events are only a measure of observed harm, whilst risk includes negative incidents that are both known and unknown. What is harm or an adverse event in the context of qualitative research? In clinical trial research, there is an established definition in the guidelines for Good Clinical Practice (GCP): any untoward medical occurrence in a patient or clinical investigation subject administered a pharmaceutical product; and which does not necessarily have to have a causal relationship with this treatment [4]. For other types of human participant research, the definition of an adverse event is somewhat ‘blurrier’. In fact, in reviewing the literature for this Editorial, I could not find an agreed definition. Many definitions were from university ethics committees. For example, consider this definition from my home institution: In Australia, in human research outside the realm of clinical trials, […] an “adverse event” is generally understood as any unintended and harmful occurrence affecting participants (or others) during the research [5]. Now that we have clearly—or perhaps not so clearly—defined adverse events, let us consider how often they occur in qualitative studies.

I performed an extensive desktop search to find literature reviews and primary studies that report on rates of harm in qualitative studies and was surprised at how little has been published on this issue. Much of the literature is focused on populations that require special consideration (often referred to as vulnerable populations), predominantly people with mental illness or who have experienced trauma and children and young people. A review of 12 studies (5 of which were qualitative) examined the risks and benefits of participating in trauma-focused research [6]. The authors reported that in all included studies, a few study participants (between 5% and 9%) reported some type of emotional distress, including feeling sad or upset, intense emotions, feeling out of control, and a worsening of mental health. Biddle et al. [7] combined data from four studies that involved interviewing people about suicide and self-harm who were invited to rate their emotional states before and after the interview and comment on the experience. A worsening of mood, because they were asked to talk about difficult issues, was reported for around a quarter of the participants. Higher rates of distress were observed in a study by Carlson et al. [8], who reported on the level of being ‘upset’ among a group of 223 psychiatric inpatients interviewed about trauma experiences. The authors reported that 66 (30%) were highly distressed by answering the interview questions. In summary, the published literature estimating the prevalence of adverse events in qualitative research is, to say the least, sparse and somewhat dated, but harm clearly does occur.

What is the situation regarding the reporting of harm in published qualitative studies? I was intrigued to examine if adverse events are reported in the qualitative papers published in *Nursing Reports*. In 2025, *Nursing Reports* published 452 documents, 30 of which were indexed in SCOPUS as qualitative studies. The analyzer tool in co-pilot was used to review the abstracts of each paper to identify if adverse events or harms were reported. Surprisingly, in this—not necessarily representative—group of qualitative papers, no authors had reported harm that occurred during the study. Browse through qualitative papers published in other leading nursing journals (or social science journals more broadly), and I am confident that you will find that harm will not be reported.

One of the reasons harm is not reported in qualitative studies may relate to the clarity of instructions provided in reporting guidelines. Reporting guidelines were introduced to enhance the completeness and transparency of research reporting [9]. When drafting a paper for submission for publication, most authors will structure their manuscript against a relevant checklist. Quite reasonably, you may assume that reporting guidelines are comprehensive; this is not correct. Indeed, several authors have been vociferously critical of the completeness of guidelines for qualitative research [10]. The two key reporting guidelines for qualitative studies are COREQ (Consolidated Standards for Reporting Qualitative Research [11]) and SQR (Standards for Reporting Qualitative Research [12]), and, upon review, it is apparent that neither make any provision for adverse events. This likely explains, at least in part, why harms are seemingly rarely reported in this type of research.

If harm is not reported in qualitative studies, how do researchers make an informed judgement as to the possible risks (and it seems there are risks) to participants when submitting an ethics application? The simple answer is that they cannot. When drafting ethics applications, investigators likely err on the side of caution—given that HRECs have a notoriously low-risk threshold—submitting their application for consideration by a full (as opposed to an expedited or low-risk review body) committee, which can be a slow and frustrating process. This also means that potential participants are told that a study has risks that are based almost exclusively on researcher conjecture and not evidence. This is not a sound basis for making an informed decision, and it likely discourages potential participants from taking part in the research. Both are important ethical issues that need consideration.

There are several ways we can address the issue of risk determination in qualitative research:

1. Further research—involving both researchers and participants involved in qualitative studies—into whether a study caused distress or other unexpected harm.
2. More considered and comprehensive reporting guidelines for qualitative research can be developed, as current guidelines are simply not fit for purpose.
3. Journal editors can instruct authors submitting papers to consider reporting unexpected events and harms that occurred while conducting qualitative research.
4. Ethical committees can more critically consider the risks associated with proposed research, considering if the identified risks are the product of excessive caution by researchers.
5. Patient and public consultation can be increased when planning research to determine the possible risks a proposed study poses to participants.

We conduct research to enhance health and wellbeing. It is important—and a foundational ethical principal—for potential participants to fully understand the risks involved in participating in qualitative research. Nurse researchers and research students need to accurately inform participants—particularly those from groups that require special consideration—of the possible risks associated with participating in qualitative research.
